# The impact of peppermint oil on the irritable bowel syndrome: a meta-analysis of the pooled clinical data

**DOI:** 10.1186/s12906-018-2409-0

**Published:** 2019-01-17

**Authors:** N. Alammar, L. Wang, B. Saberi, J. Nanavati, G. Holtmann, R. T. Shinohara, G. E. Mullin

**Affiliations:** 1 The Division of Gastroenterology and Hepatology, The Johns Hopkins University School of Medicine, 600 N. Wolfe Street, Baltimore, MD 21287 USA; 2 King Khalid University Hospital, King Saud University, P.O. Box 2925, Riyadh, 11461 Saudi Arabia; 3 The Johns Hopkins School of Public Health, Baltimore, MD USA; 4 The Division of Liver Medicine, Gastroenterology, The Mount Sinai Hospital, New York, NY USA; 5 Department of Gastroenterology & Hepatology, Princess Alexandra Hospital, Brisbane, University of Queensland, QLD, Brisbane, Australia; 6 Department of Biostatistics, Epidemiology, & Informatics, Perelman School of Medicine, University of Pennsylvania, Philadelphia, PA USA

**Keywords:** Irritable bowel syndrome, IBS, Peppermint oil, Global symptom relief, Abdominal pain, Meta-analysis, PRISMA

## Abstract

**Background:**

Peppermint oil (PO) has intrinsic properties that may benefit patients with irritable bowel syndrome (IBS) symptoms. The study objective was to determine the effect of peppermint oil in the treatment of the IBS.

**Methods:**

We systematically searched MEDLINE (PubMed), Cochrane Central Register of Controlled Trials (Cochrane CENTRAL), ClinicalTrials.gov, EMBASE (Ovid), and Web of Science for randomized controlled trials (RCTs) of PO for IBS. We appraised the eligible studies by the Cochrane risk of bias tool. We performed random-effects meta-analysis on primary outcomes including global improvement in IBS symptoms and abdominal pain. A PRISMA-compliant study protocol is registered in PROSPERO Register [2016, CRD42016050917].

**Results:**

Twelve randomized trials with 835 patients were included. For global symptom improvement, the risk ratio (RR) from seven RCTs for the effect of PO (*n* = 253) versus placebo (*n* = 254) on global symptoms was 2.39 [95% confidence interval (CI): 1.93, 2.97], *I*^*2*^ = 0%, z = 7.93 (*p* < 0.00001). Regarding abdominal pain, the RR from six RCTs for the effect of PO (*n* = 278) versus placebo (*n* = 278) was 1.78 [95% CI: 1.43, 2.20], *I*^*2*^ = 0%, z = 5.23 (*p* < 0.00001). Overall, there were no differences in the reported adverse effects: PO (32 events, 344 total, 9.3%) versus placebo (20 events, 327 total, 6.1%) for eight RCTs; RR 1.40 [95% CI: 0.87, 2.26] I^2^ = 0%, z = 1.39 (*p* = 0.16). The number needed to treat with PO to prevent one patient from having persistent symptoms was three for global symptoms and four for abdominal pain.

**Conclusions:**

In the most comprehensive meta-analysis to date, PO was shown to be a safe and effective therapy for pain and global symptoms in adults with IBS.

**Electronic supplementary material:**

The online version of this article (10.1186/s12906-018-2409-0) contains supplementary material, which is available to authorized users.

## Background

The irritable bowel syndrome (IBS) is a chronic, functional gastrointestinal syndrome characterized by relapsing abdominal pain and altered bowel habits, with either predominant symptoms of diarrhea (IBS-D), constipation (IBS-C), both (IBS-M), or undetermined (IBS-U), and is categorized according to the Rome IV criteria [1]. As a common digestive tract disorder, IBS affects an estimated 5–15% of Western populations [2]. Lovell and Ford conducted a meta-analysis of the world’s literature and reported that, on a global scale, IBS is seen predominantly in females, and the age of onset is typically under 50 years-of-age [3]. In their research, Lovell and Ford found the global prevalence of IBS to be 11.2% (95% confidence interval [CI], 9.8–12.8%) [3]. IBS accounts for a significant number of annual visits to primary care physicians, health-care utilization, quality of life, and adverse economics owing to absenteeism from work [4].

The pathophysiology of IBS is complex and involves an interaction of various factors, which includes, but is not limited to, genetic predisposition, gut-brain axis, visceral sensitivity, gastrointestinal motility, gut dysbiosis, neurotransmitters, food reactions, intestinal permeability, bile acids, inflammatory mediators, early-life stressors, psychosocial maladaptation, and somatization, among others [5]. IBS patients with mild and intermittent symptoms usually benefit from lifestyle and dietary modification, which includes a diet low in fermentable oligo-, di-, and monosaccharides and polyols (FODMAPs) [6]; and in some cases, lactose and gluten avoidance [7]. Smooth muscle relaxants and antispasmodics can also be used to help with IBS symptoms, especially abdominal pain and bloating [8].

Peppermint oil (PO) (*Mentha Piperita*) is a naturally-occurring carminative herb containing monoterpene compounds that target the pathophysiology of IBS. PO contains L-menthol, which blocks calcium channels in smooth muscle, thus producing antispasmodic effects on the gastrointestinal tract [9]. PO possesses antimicrobial, anti-inflammatory, antioxidant, immunomodulating, and anesthetic activities, all of which may be relevant for the treatment of IBS [10–12]. Several case reports, observational studies, and randomized clinical trials (RCTs) with methodological inconsistencies and heterogeneous outcomes have been reported since the research conducted by Rees et al. in 1979 [8, 13–20]. Earlier systematic reviews of RCTs of PO for IBS treatment revealed trial design flaws (e.g., no washout period for crossover trials), short follow-up duration, and conflicting trial results [14, 21]. Some more recent systematic reviews of RCTs of PO for IBS treatment were limited in the lack of evidence for adverse events [8, 18]. In addition, the risk-benefit profile of PO has been evolving as new RCTs continue to arise.

In 2016, Cash et al. reported the findings of a 4-week double blinded, placebo controlled RCT which tested a novel, proprietary, enteric-coated peppermint formulation (IBgard®) for its potential efficacy in reducing IBS symptoms in 72 patients with IBS-M or IBS-D [22]. The specialized enteric-coating utilized in their trial consisted of a solid-state matrix that was triple-coated and designed to deliver PO with sustained release to the small intestine with fewer potential adverse effects. After 24 h. of treatment, there was a reduction in the total IBS symptom score over baseline (mean change − 0.55, SD ± 0.613) vs. placebo (mean change − 0.27, SD ± 0.342) (*p* = 0.0092). At trial completion, there was a 40% reduction in the total IBS symptom score in the PO group compared to baseline (mean change − 1.16, SD ± 0.807) vs. 24.3% (mean change − 0.70, SD ± 0.737) with placebo (*P* = 0.0246). There was an increased improvement in both multiple and individual gastrointestinal symptoms, as well as in severe or unbearable symptoms compared to the placebo.

Given the recent findings by Cash et al. [22] and the potential limitations of previous meta-analyses, we conducted a systematic review and meta-analysis of available RCTs to determine the effect of peppermint oil in reducing the abdominal pain and global symptoms of irritable bowel syndrome and to evaluate the possible side effects of PO as compared to the placebo.

## Methods

### Identification and retrieval of primary studies

We conducted this systematic review and meta-analysis as per the PRISMA guidelines (i.e., the preferred reporting items of systematic reviews and meta-analysis) [23]. An experienced medical informationist (JN) developed and executed the research strategy in collaboration with the co-authors. There were no restrictions placed on publication dates. A preliminary search was executed on October 10, 2016, and repeated on October 10, 2017, and April 11, 2018, using the following databases: MEDLINE (PubMed), Cochrane Central Register of Controlled Trials (Cochrane CENTRAL), ClinicalTrials.gov, EMBASE (Ovid), and Web of Science. Controlled vocabulary terms for each concept were identified and combined with keyword synonyms. Web of Science was searched using keyword terms only (please see Additional file 1. Medical Literature Search Results for full search strategies). All results were downloaded to Endnote X8 (Thompson and Reuters, Philadelphia, Pennsylvania) and duplicate citations were identified and removed. The protocol is registered in PROSPERO Register [2016:CRD42016050917; (http://www.crd.york.ac.uk/PROSPERO/display_record.asp?ID=CRD42016050917)].

### Study selection and data extractions

The titles and abstracts of the studies were carefully reviewed by two of the authors (GM, NA) independently to include RCTs that evaluated the influence of enteric-coated PO on IBS, based on the inclusion and exclusion criteria (Table 1). When there was a disagreement, a third reviewer (BS) determined whether the study qualified for inclusion. We also reviewed the bibliography of prior meta-analyses, review articles, and studies that underwent full-text screening for additional studies (reverse snowballing) to maximize the yield [24].

**Table 1:** Selection criteria for inclusion and exclusion

	Criteria
Inclusion	1. Randomized placebo-controlled trials comparing peppermint oil and placebo for irritable bowel syndrome with a minimum treatment duration of 2 weeks. 2. Adult patients with irritable bowel syndrome as diagnosed using any of the following criteria for IBS: Manning, Rome I, II, III, IV diagnostic criteria.
Exclusion	1. Non-randomized trials; observational studies such as cohort study, cross-sectional study, etc.. 2. Patients having organic disease or or did not have organic disease excluded. 3. Treatment duration of less than 2 weeks. 4. Studies with inadequate data.

Once the articles met the criteria, the full text was reviewed and data extraction performed by four independent reviewers (GM, BS, GH, LW) based on data quality, sufficiency, and relevance. Disagreements were resolved by a third reviewer to reach a consensus. Our primary outcomes are proportion of patients with improvement in global symptoms and proportion of patients with improvement in abdominal pain. Extracted data included last name of the first author, year of publication, country of origin, study setting, demographic information of patients, publication year, population, sample size, study design, subtype(s) of IBS (if specified), criterion used for the IBS diagnosis, peppermint oil dose, preparation of peppermint oil, and patients enrolled and completed, and quantitative results. For RCTs with cross-over design, we only extracted data from the first stage before the wash-out period to account for intra-patient correlation of outcomes.

### Risk of bias, quality assessment, and data synthesis

We used the modified Cochrane Collaboration’s risk of bias assessment tool for RCTs. Bias was assessed as a judgment (high, low, or unclear) for individual elements from five domains (selection, performance, attrition, reporting, and other) [25]. Any disagreements were then discussed with a third reviewer (BS) with an agreement to be reached by consensus [25]. The Grading of Recommendations Assessment, Development, and Evaluation (GRADE) analysis was utilized to rate the evidence of our review, whereby very low = 1, low = 2, moderate = 3, high = 4. The strength of recommendations were 1 (strong) or 2 (weak) [26].

### Statistical analysis

We pooled the results from included studies by random-effects meta-analysis with inverse variance weighting to determine the risk ratio (RR) and the 95% confidence interval (95% CI) for each outcome in RevMan 5.3.5 [27]. Q statistics, I-squared (*I*^2^), and tau-squared (τ^2^) were calculated to assess statistical heterogeneity. For Q statistics, a critical value of 0.1 was used to determine statistical significance. We considered an *I*^2^ greater than 0.75 as a cutoff for considerable heterogeneity across studies. We planned to use funnel plots and Egger’s test [28] to examine publication bias if the number of studies for an outcome is larger than ten. We conducted sensitivity analyses by removing studies with a high risk of bias per the Cochrane risk of bias tool.

## Results

### Study selection

**Fig. 1:**
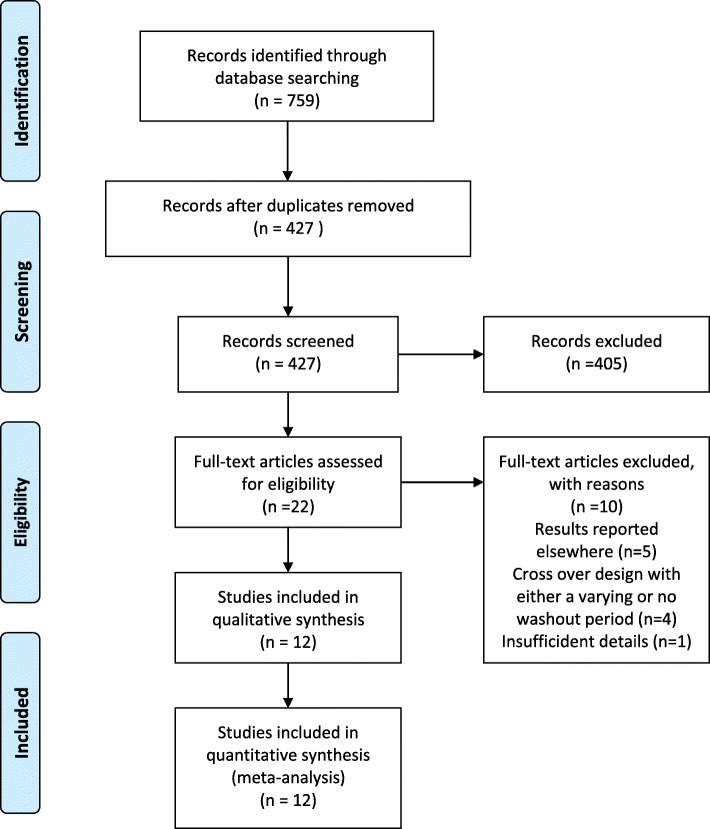
PRISMA Flow Diagram for Study Selection. PRISMA flowchart illustrating the process for the selection of the included articles for the systematic review and for the data synthesis of the randomized controlled clinical trials of enteric-coated peppermint oil versus placebo in patients with irritable bowel syndrome (IBS). Inclusion: Adult patients (18 years or greater) with IBS as diagnosed using any of the following criteria for IBS: Manning Criteria, Rome I, II, III, IV diagnostic criteria who were randomized to enteric-coated peppermint oil or placebo for a minimum of two weeks. Exclusion: Patients having an organic disease or who had not had an organic disease were excluded. Non-randomized trials; observational studies such as cohort study, cross-sectional study, etc.. A detailed evaluation of the articles by at least two independent reviewers (total of three) assessed the sufficiency of data and relevance to the topic. Seven hundred and fifty-nine articles were identified using PubMed (*n* = 102)/EMBASE (*n* = 396)/Cochrane (*n* = 60)/Web of Science (*n* = 201) search engines. After de-duplication, 427 records were screened and 22 deemed suitable for full-text review. Ten articles were eliminated, thus leaving twelve for qualitative and data synthesis

A literature search conducted from inception to April 11, 2018, identified 759 studies. After duplicates were removed, a total of 427 studies remained for a review of titles and abstracts, from which 22 trials were identified that underwent full text screening. A total of ten studies were excluded (Additional file 2**:** Table of Excluded Studies), and twelve randomized studies (835 patients) that met the inclusion criteria were identified and underwent systematic review and data synthesis. A flow diagram of the study selection is summarized in Fig. 1.

### Study characteristics

The included studies were published over five decades, from 1979 to 2016. Patients from Asia, Europe, and North America were recruited. Studies were of varied sample sizes, from 18 to 178. The settings of most trials were teaching hospitals. Most studies were double-blind parallel group RCTs with follow-up durations ranging from 3 weeks to 12 weeks. Cross-over design were observed in three studies. Table 2 summarizes the characteristics of the included studies. Of note, among the twelve included studies, there was significant variation in the use of validated tools for the diagnosis of IBS. Alam et al. [29], Cappello et al. [30], Capanni et al. [31], and Merat et al. [32] utilized the Rome II criteria. Cash et al. [22] used the Rome III criteria and found a statistically significant benefit for PO relative to placebos for the global improvement of IBS symptoms. Dew et al. [33] conducted a double-blind cross-over study with a washout period defined by the recurrence of active symptoms, however, they failed to utilize any validated inclusion criteria. Lech et al. [34] failed to utilize any validated inclusion criteria, though they did find a significant benefit for PO relative to placebo for improvements in abdominal pain. Liu et al. [35] failed to utilize any validated inclusion criteria, though they did find a significant benefit for PO relative to placebo for the improvement of abdominal pain. Rees et al. [20], Schneider et al. [36], Weiss et al. [37], and Carling et al. [38] also failed to mention the use of validated inclusion criteria.

**Table 2:** Characteristics of Included Studies

Year	Author	Country	Design	Setting	N Enrolled	N Completed	Duration of therapy
2013	Alam	Bangladesh	Double-Blind RCT	University Single-Center	74	65	6 weeks
2016	Cash	USA	Double-Blind RCT	Multicenter	72	70	4 weeks
2005	Capanni	Italy	Double-Blind RCT	University Single-Center	178	173	12 weeks
2007	Cappello	Italy	Double-Blind RCT	University Single-Center	57	50	4 weeks
1989	Carling	Sweden	Double-blind Cross Over 3-arm RCT 1-Week Washout	2 University Centers	40	38	2 weeks
1984	Dew	Wales	Double-Blind Cross Over Washout Period	Multicenter	29	29	2 weeks
1988	Lech	Dutch	Double-Blind RCT	University Single-Center	47	42	4 weeks
1997	Liu	China	Double-Blind RCT	University Single-Center	110	101	4 weeks
2009	Merat	Iran	Double-blind RCT	University Single- Center	90	60	8 weeks
1979	Rees	UK	Double-Blind Cross Over Washout Period Defined by Recurrence of Active Symptoms	University Single- Center	18	16	3 weeks
1990	Schneider	USA	Double-blind Cross Over RCT 2-Week Washout	University Single- Center	60	47	6 weeks
1988	Weiss	Germany	Single blinded, RCT	Hospital, single center	60	46	3 weeks

### Risk of bias assessment

**Fig. 2:**
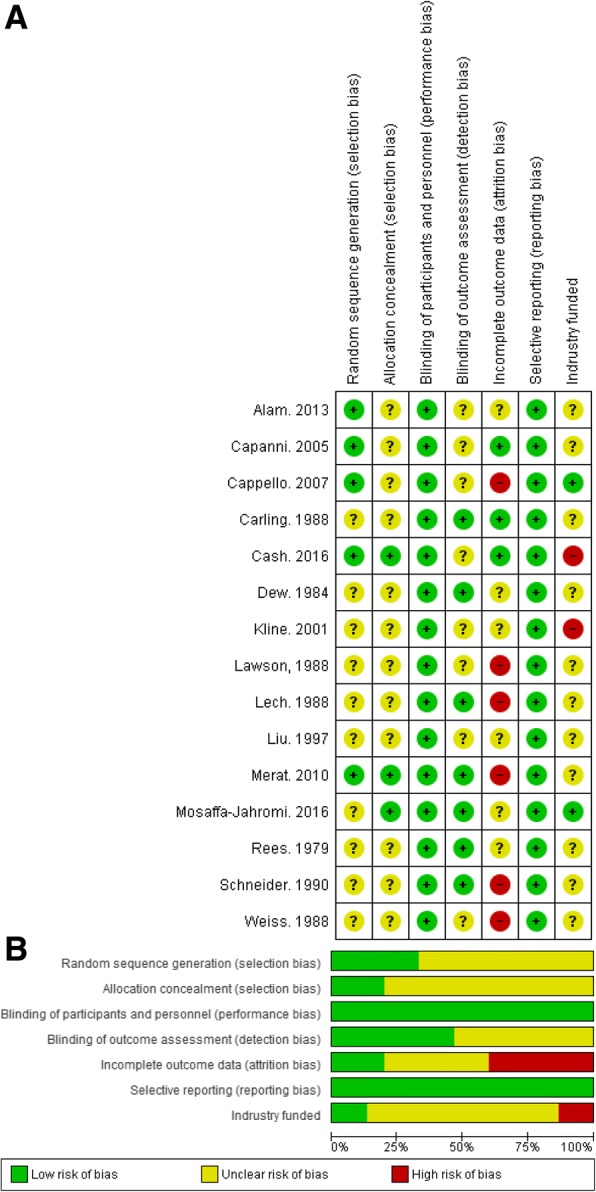
**a**-**b**. Risk of Bias Assessment Using Cochrane the Collaboration’s Tool. The included studies were evaluated for methodological flaws using the Cochrane Collaboration’s risk of bias assessment tool. [25] **a** illustrates the risk of bias for each study. Studies were indexed by the last name of the first author and year of publication. Seven domains of risk of bias were assessed for each study, including random sequence generation, allocation concealment, blinding of participants and personnel, blinding of outcome assessment, incomplete outcome data, selective reporting, and industry funded. , , denote low, unclear, and high risk of bias, respectively. Six of the 12 studies were assessed as having high risk of attrition bias and two studies were funded by industry (high risk of bias). Ten of the 12 included studies did not report random sequence generation and allocation concealment (unclear risk of selection bias). In contrast, the blinding of participants and personnel were well performed (low risk of performance bias in seven of the 12 included studies). Selective reporting was not observed in any studies (low risk if bias). Figure 2b shows the overall risk of bias by domain: the risk of bias is displayed as low risk (green, +), unclear (yellow,?), or high risk (red, −)

Incomplete outcome data was the most concerning problem observed in the included studies (Fig. 2). Six out of 12 studies were assessed as having high risk of attrition bias as these studies had over 10% loss to follow-up and dealt with missing data by excluding those patients from final analyses. Two studies were funded by industry and were assessed as having high risk of bias due to conflicts of interest. In addition, random sequence generation and allocation concealment were not reported (unclear risk of selection bias) in many studies. In contrast, the blinding of participants and personnel were well performed in all studies (low risk of performance bias). The selective reporting was not observed in any studies (low risk of reporting bias).

### Meta-analysis

**Fig. 3:**
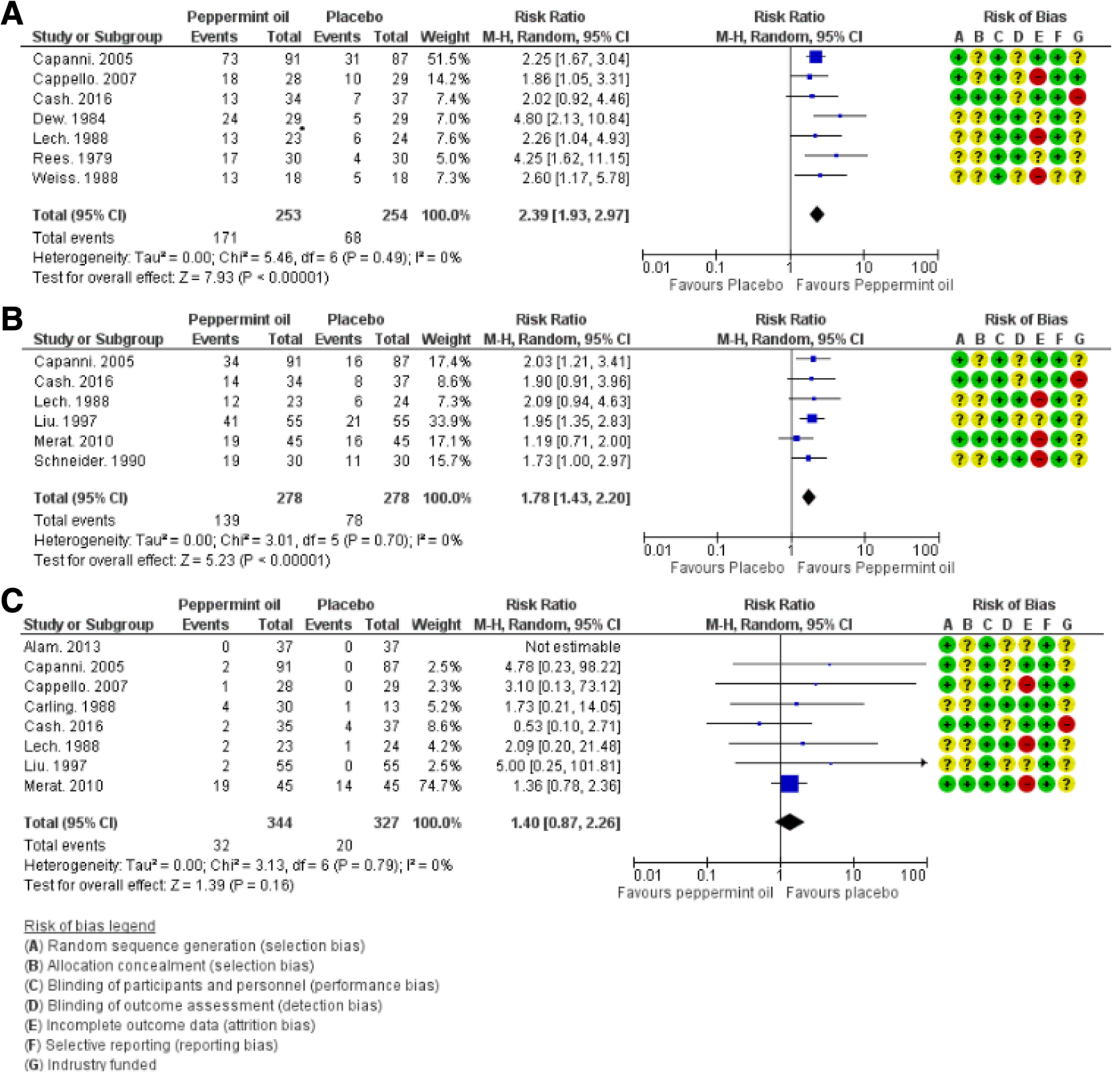
**a**-**c**. Forrest Plots of Meta-analysis of Enteric-Coated Peppermint Oil for the treatment of Irritable Bowel Syndrome. The results of our meta-analysis of the randomized controlled trials of enteric-coated peppermint oil (PO) versus placebo for the irritable bowel syndrome (IBS) are shown in **a-c**. **a** illustrates the results of the meta-analysis of treatment outcomes for seven included studies for the global improvement of IBS symptoms vs. placebo [20, 22, 31, 33–35, 37]. **a**. illustrates the risk ratio (RR) for seven RCTs for the effect of enteric-coated PO (*n* = 253) versus placebo (*n* = 254) on global symptoms was 2.39 [95% confidence interval (CI): 1.93, 2.97], *I*^*2*^ = 0%, z = 7.93 (*p* < 0.00001). **b**. displays the results of the meta-analysis of the reported treatment outcomes for the improvement in abdominal pain vs. placebo for six included studies [22, 31, 32, 34, 36]. The RR for six RCTs for the effect of enteric-coated PO (*n* = 278) versus placebo (*n* = 278) on abdominal pain was 1.78 [95% CI: 1.43, 2.20], *I*^*2*^ = 0%, z = 5.23 (*p* < 0.00001). **c** illustrates the results of the meta-analysis of eight included studies [22, 29–32, 34, 35, 38] of the reported adverse effects in IBS subjects using EPO (32 events, 344 total, 9.3%) versus placebo (20 events, 327 total, 6.1%); RR 1.40 [95% CI: 0.87, 2.26] *I*^*2*^ = 0%, z = 1.39 (*p* = 0.16). Figure 3a-c show their corresponding risk of bias analysis. (A) Random sequence generation (selection bias); (B) Allocation concealment (selection bias); (C) Blinding of participants and personnel (performance bias); (D) Blinding of outcome assessment (detection bias); (E) Incomplete outcome data (attribution bias); (F) Selective reporting (reporting bias); (G) Industry funded. The risk of bias is displayed as low risk (green, +), unclear (yellow, ?), or high risk (red, −)

Seven studies reported treatment outcomes for the global improvement of IBS symptoms [20, 22, 31, 33–35, 37] (Fig. 3a). The risk ratio (RR) for seven RCTs for the effect of PO (*n* = 253) versus placebo (*n* = 254) on global symptoms was 2.39 [95% confidence interval (CI): 1.93, 2.97], *I*^2^ = 0%, z = 7.93 (*p* < 0.00001) (Fig. 3a). The number of patients needed to be treated with peppermint oil versus the placebo to induce a global improvement of IBS symptoms was three (Table 3). No statistically significant heterogeneity was detected for this comparison (*τ*^2^ = 0.00, *X*^2^ = 5.29, *P* = 0.51, *I*^2^ = 0%) (Table 3).

**Table 3:** Summary of Findings. Peppermint Oil vs. Placebo for the Treatment of IBS

Patient or Population: Patients with Active IBS						
Settings: Outpatients						
Intervention: Enteric-coated Peppermint Oil Capsules vs. Placebo						
Outcomes	Illustrative Comparative Risk*					
	Assumed risk	Corresponding risk				
	Control (per 1000)	Peppermint Oil vs. Placebo (per 1000)	Relative Risk (95% CI)	No. Participants (Studies)	Quality of Evidence (GRADE)	NNT (95% CI)
Global improvement in IBS symptoms	250†	598 (483 to 743)	2.39 (1.93–2.97)	507 (7)	⨁⨁⨁⨁‡ High	3 (2–4)
Improvement in abdominal pain	303†	539 (433 to 666)	1.78 (1.43–2.20)	556 (6)	⨁⨁⨁◯§ Moderate	4 (3–6)
Adverse events	21†	29 (18 to 47)	1.40 (0.87–2.26)	671 (8)	⨁⨁◯◯ǁ Low	125 (29-∞)

GRADE Working Group grades of evidence. *High quality*: further research is very unlikely to change our confidence in the estimate of effect. *Moderate quality*: further research is likely to have an important impact on our confidence in the estimate of effect and may change the estimate. *Low quality*: further research is very likely to have an important impact on our confidence in the estimate of effect and is likely to change the estimate*. Very low quality*: we are very uncertain about the estimate

*The basis for the assumed risk is the median control group risk across studies) . The corresponding risk (and its 95% CI) is based on the assumed risk in the comparison group and the relative effect of the intervention (and its 95% CI)

†Control group risk estimates come from the control arm of meta-analysis, based on included trials

⨁⨁⨁⨁‡ High: downgraded on risk of bias, upgraded on large magnitude of effect

⨁⨁⨁◯§ Moderate: downgraded on risk of bias

⨁⨁◯◯ǁ Low: downgraded on risk of bias and imprecision

CI indicates confidence interval

Six studies reported treatment outcomes of improvement of abdominal pain [22, 31, 32, 34, 36] (Fig. 3b). The RR for six RCTs for the effect of PO (*n* = 278) versus placebo (*n* = 278) on abdominal pain was 1.78 [95% CI: 1.43, 2.20], *I*^*2*^ = 0%, z = 5.23 (p < 0.00001) (Fig. 2b). The number of patients that needed to be treated with peppermint oil versus the placebo to improve abdominal pain was four (Table 3). No statistically significant heterogeneity was detected for this comparison (*τ*^2^ = 0.00, *X*^2^ = 3.01, *P* = 0.70, *I*^*2*^ = 0%) (Table 3).

Of the studies included, overall, only a few adverse events were reported. One study reported no adverse events [25]. Most of the reported adverse events were mild and transient. The adverse events included: heartburn [13, 15, 22, 23, 29, 30, 35], dry mouth [13, 23], belching [23], peppermint taste [15], rash [29], dizziness [23], headache [23], and less frequent increased appetite [23]. There was no statistically significant difference in reported adverse effects in IBS subjects using PO (32 events, 344 total, 9.3%) versus placebo (20 events, 327 total, 6.1%); RR 1.40 [95% CI: 0.87, 2.26], *I*^*2*^ = 0%, z = 1.39 (*p* = 0.16) (Fig. 3c).

The planned funnel plots and Egger’s test for publication bias was not applicable as the number of studies for each outcome is less than ten because test power is usually too low to distinguish chance from real asymmetry [28, 39]. The sensitivity analyses excluding studies with high attrition bias provided similar results to our primary analyses.

The GRADE criteria were used to assess the overall quality of the evidence reported (Table 3) [26]. The global improvement in IBS symptoms outcomes was upgraded to high because of the large magnitude of the effect. The outcome of improvement of abdominal pain was downgraded to moderate because of the risk of bias. The outcome “adverse events” was downgraded to “low quality” because of the risk of bias and imprecision (Table 3).

## Discussion

In this systematic review, we assessed the largest cohort of RCTs published over five decades involving twelve randomized clinical trials with 835 IBS patients from around the world. Overall, treatment with PO significantly improves abdominal pain and global symptoms of IBS. The available data are also consistent with a good safety profile. The strength of our findings is reflected by the large effect size of PO over placebo in the improvement of abdominal pain and global symptoms and by the low heterogeneity across included studies.

The first systematic review of RCTs of PO for the treatment of IBS was published by Pitter and Ernst in 1998, which included eight randomized trials involving 295 patients with seven of the eight trials not using the accepted clinical features of IBS [14]. The researchers performed a quantitative synthesis of five double-blind, placebo-controlled RCTs involving 265 participants [20, 33, 34, 38, 40]. Four of the five RCTs had a Jadad methodological quality score of three, with no RCTs scoring the maximum of five points [41]. Overall, the results demonstrated that PO was effective for the improvement of global symptoms in IBS (*p* < 0.001). However, two of the five studies showed no difference when using a placebo in IBS symptom improvement, and overall, there was a significant variation between the placebo responses across the five studies (13–52%, *p* < 0.01). No definitive conclusion could be drawn owing to the low quality of the primary studies, the overrepresentation of short-term (< 1 month duration) studies, and the use of cross-over designs without washout periods in four of the five RCTs. The authors acknowledged that the results of their meta-analysis needed to be interpreted with caution due to the mentioned methodological flaws in the included studies. We also observed that six of the eight trials included in the Pittler and Ernst review [14] had treatment periods of one month or less. The studies included in this meta-analysis had treatment periods of two to twelve weeks, with seven studies being four weeks or greater, and found a significant benefit for PO relative to placebo for the improvement of abdominal pain and global IBS symptoms.

In 2004, Lesbros-Pantoflickova et al. performed a meta-analysis of the available pharmacological treatments for the irritable bowel syndrome, which included PO [21]. The authors included five studies [20, 33, 34, 38, 40], with four having a Jadad score ≥ 3 [33, 38, 40]. Overall, the odds ratio (OR) of the five included studies favored PO for global symptoms over the placebo [OR 3.6, 95 CI% 2.2–6.0]. Lesbros-Pantoflickova et al.’s systematic review and meta-analysis lacked several methodological details and improperly concluded that Pittler’s meta-analysis failed to demonstrate a beneficial effect for PO vs. placebo for improving IBS symptoms [14].

In 2008, Ford et al. reported the results of a qualitative and quantitative synthesis of the available studies for the effect of fiber, antispasmodics, and PO in the treatment of IBS [8]. Four of the included studies had a Jadad score ≥ 3 [30, 31, 34, 35] with a total of 392 participants to evaluate the effect of PO versus placebo on IBS symptoms. Ford et al. excluded the cross-over trials included by Pittler and Ernst [14]. They reported that the relative risk of persistent symptoms was 43% less with PO (52/197; 26%) when compared to placebo (127/195; 65%) (relative risk, 0.43) without any significant heterogeneity between studies (*I*^*2*^ = 31.1%, *P* = 0.23). The number needed to treat (NNT) with peppermint oil to prevent one patient from having persistent symptoms was 2.5 (2.0–3.0). The methodological details of the selection criteria and extraction were provided, however, the criteria to define symptom improvement was heterogeneous and included pain and/or global symptom improvement [8]. We separately analyzed the ability of PO to improve abdominal pain and global IBS symptoms. A limited risk of bias showed that all studies lacked concealed allocation. It is worth mentioning that a meta-analysis was not conducted on the side-effect data as only three trials reported adverse events.

In 2011, Ruepert et al. published the results of their systematic review and meta-analysis on the effectiveness of antispasmodics, antidepressants, and bulking agents in IBS, which included the randomized controlled trials of PO versus placebo [17]. PO was shown to improve global symptoms; risk ratio was 2.25 [1.70–2.98] in two studies with 225 patients [31, 34]. PO also improved the IBS symptom score vs. placebo; risk ratio was 1.94 [1.09–3.46] in three studies with 269 patients [30, 31, 42]. Their analysis of spasmolytics for the relief of abdominal pain demonstrated the superiority of PO versus placebo; risk ratio was 2.15 [1.54, 3.00] in one trial of 101 patients [35].

The most recent meta-analysis by Khanna et al. (2014) evaluated 726 patients [19] from nine [29, 30–32, 34–37, 43] included studies. Global IBS symptom improvement was reported to be greater for PO versus placebo (5 studies, 392 patients, relative risk 2.23; 95% confidence interval, 1.78–2.81), and likewise for improvement in abdominal pain (5 studies, 357 patients, relative risk 2.14; 95% confidence interval, 1.64–2.79). Khanna et al.’s pooled analysis of seven studies and 474 patients reported that IBS patients treated with PO, as compared to the placebo, were more likely to experience an adverse event, such as heartburn, which tended to be mild and transient [19].

In 2018 Ford et al. published a systematic review of RCTs using medical, psychological and nutritional therapies for IBS as an updated monograph for the American College of Gastroenterology [44]. The 2014 version included five RCTs of PO versus placebo for IBS [45]. The primary outcome of improved IBS outcome was not defined according to global symptoms versus pain relief. The search terms were merged for a number of interventions (i.e. fiber, diet) with colpermin and peppermint oil being utilized to identify RCTs using PO for IBS. Seven RCTs involving 634 patients were included and the pooled analysis showed benefit for PO over placebo (RR-0.54; 95% CI 0.39–0.76). The number needed to treat in order for one patient to benefit was four but the endpoint of IBS improvement was not defined and heterogeneity was high (*I*^*2*^ = 73%, *P* = 0.001). Pooled data on adverse events from six studies did not show a difference between PO and placebo. One of the two new included studies was a comparative study of peppermint oil and anise oil to placebo [46] with a visual analog scale and quality of life as primary and secondary endpoints. For these reasons, this study was excluded from our analysis.

Overall, we evaluated 835 adult patients from twelve studies that met the inclusion criteria. Improved global IBS symptomatology was greater for PO when compared to placebo, as well as for abdominal pain. We included studies excluded by Khanna et al. [20, 22, 33, 38] and excluded their included study on pediatric IBS [47]. Unlike Khanna et al., we did not detect a difference in the adverse events reported in IBS subjects using PO versus placebo. Our risk of bias analysis also differed from that of Khanna et al., as we found a high risk of bias for Cash et al. [22] for industry funding and attrition bias for Cappello et al. [30], Rees et al. [20], and Weiss et al. [37], which shall bring necessary caution to result interpretation. In our study, the number needed to treat with PO to prevent one patient from having persistent symptoms was three for global symptoms and four for abdominal pain.

## Conclusions

Enteric-coated peppermint oil is a safe and effective therapy for the relief of abdominal pain and global symptoms and in adults with IBS.

## Additional files

Additional file 1: Table of Medical Literature Search Results for Randomized Controlled Trials of Enteric-Coated Peppermint Oil for the Irritable Bowel Syndrome. We searched the medical literature for randomized controlled trials of enteric-coated peppermint oil for the treatment of the irritable bowel syndrome. Our search methodology yielded 102 reports in PubMed, 396 in Embase, 201 in Web of Science, and 60 in the Cochrane library. (PDF 265 kb)

Additional file 2: Table of Excluded Studies. Ten of twenty-two included randomized controlled trials (RCTs) were eliminated for reasons shown. Five studies for data that was shown in other publications, four RCTs were cross-over trials without sufficient washout period, and an RCT was permitted rescue medication without sufficient data to permit analysis. (PDF 369 kb)

## Abbreviations

CI: Confidence interval
EPO: Enteric-coated peppermint oil
FODMAPs: Fermentable oligo-, di-, and monosaccharides and polyols
IBS: Irritable bowel syndrome
IBS-C: Irritable bowel syndrome: constipation predominant
IBS-D: Irritable bowel syndrome: diarrhea predominant
IBS-M: Irritable bowel syndrome: mixed
IBS-U: Irritable bowel syndrome: undetermined
NNT: Number needed to treat
PO: Peppermint oil
RCTs: Randomized clinical trials
RR: Risk ratio
TISS: Total IBS symptom score
