# Supplementary material for: The impact of peppermint oil on the irritable bowel syndrome: a meta-analysis of the pooled clinical data
Source: BMC Complement Altern Med. 2019 Jan 17;19:21. doi: 10.1186/s12906-018-2409-0 (PMC6337770; doi:10.1186/s12906-018-2409-0)
Supplement: Supplementary file 1 — Table of Medical Literature Search Results for Randomized Controlled Trials of Enteric-Coated Peppermint Oil for the Irritable Bowel Syndrome. We searched the medical literature for randomized controlled trials of enteric-coated peppermint oil for the treatment of the irritable bowel syndrome. Our search methodology yielded 102 reports in PubMed, 396 in Embase, 201 in Web of Science, and 60 in the Cochrane library. (PDF 265 kb) [file 12906_2018_2409_MOESM1_ESM.pdf]

Additional File 1. Table of Medical Literature Search Results for Randomized Controlled Trials of Enteric-Coated Peppermint oil for the Irritable Bowel Syndrome.

(PubMed) (1966-4/11/2018) (102 results)

#1. ("oleum menthae piperitae"[tw] OR "peppermint oil"[Supplementary Concept] OR "peppermint essentia[illegible] "peppermint oil"[tw] OR mintoil[tw] OR colpermin[tw] OR "oil of peppermint"[tw] OR 8006-90-4[RN] OR "m[illegible] oil"[tw] OR mintec[tw])

#2. ("Irritable Bowel Syndrome"[mh] OR "irritable bowel syndrome"[tw] OR "irritable bowel syndromes"[tw] OR "Mucous Colitis"[tw] OR "spastic colon"[tw] OR "irritable colon"[tw] OR "functional bowel"[tw] OR "colonic disease"[tw] OR "colonic diseases"[tw] OR IBS[tw] OR "gastrointestinal syndrome"[tw] OR "gastrointestinal syndromes"[tw] OR fgid[tw] OR "functional gastrointestinal disorders"[tw] OR "Colon spasm"[tw] OR "Irritable colon syndrome"[tw] OR "spastic colitis"[tw] OR "unstable colon"[tw])

#3. #1 AND #2

Embase (Wiley) (1980 – 4/11/2018) (396 results)

#1. ('Peppermint oil'/exp OR 8006-90-4:rn OR 'oleum menthae piperitae':ti,ab OR 'peppermint oil':ti,ab OR 'peppermint essential oil':ti, ab OR mintoil:ti,ab OR colpermin:ti,ab OR 'oil of peppermint':ti,ab OR 'mentha piperita oil':ti,ab OR mintec:ti,ab)

#2. ('irritable colon'/exp OR 'irritable bowel syndrome':ti,ab OR 'irritable bowel syndromes':ti,ab OR 'Mucous Colitis':ti,ab OR 'spastic colon':ti,ab OR 'irritable colon':ti,ab OR 'functional bowel':ti,ab OR 'colonic disease':ti,ab OR 'colonic diseases':ti,ab OR IBS:ti,ab OR 'gastrointestinal syndrome':ti,ab OR 'gastrointestinal syndromes':ti,ab OR fgid:ti,ab OR 'functional gastrointestinal disorders':ti,ab OR 'Colon spasm':ti,ab OR 'Irritable colon syndrome':ti,ab OR 'spastic colitis':ti,ab OR 'unstable colon':ti,ab)

#3. #1 AND #2

Additional File 1. Medical Literature Search Results for Randomized Controlled Trials of Enteric-Coated Peppermint oil for the Irritable Bowel Syndrome.

Web of Science (1900 – 4/11/2018) (201 results)

#1 ts=("Peppermint oil" OR "oleum menthae piperitae" OR "peppermint essential oil" OR mintoil OR colpermin OR "oil of peppermint" OR "mentha piperita oil" OR mintec)

#2 ts=("irritable colon" OR "irritable bowel syndrome" OR "irritable bowel syndromes" OR "Mucous Colitis" OR "spastic colon" OR "irritable colon" OR "functional bowel" OR "colonic disease" OR "colonic diseases" OR IBS OR "gastrointestinal syndrome" OR "gastrointestinal syndromes" OR fgid OR "functional gastrointestinal disorders" OR "Colon spasm" OR "Irritable colon syndrome" OR "spastic colitis" OR "unstable colon")

#3. #1 AND #2

Cochrane Library (60)

#1 MeSH descriptor: [Irritable Bowel Syndrome] explode all trees

#2 "irritable colon" or "irritable bowel syndrome" or "irritable bowel syndromes" or "Mucous Colitis" or "spastic colon" or "irritable colon" or "functional bowel" or "colonic disease" or "colonic diseases" or IBS or "gastrointestinal syndrome" or "gastrointestinal syndromes" or fgid or "functional gastrointestinal disorders" or "Colon spasm" or "Irritable colon syndrome" or "spastic colitis" or "unstable colon":ti,ab,kw

#3 #1 or #2

#4 "Peppermint oil" or "oleum menthae piperitae" or "peppermint essential oil" or mintoil or colpermin or "oil of peppermint" or "mentha piperita oil" or mintec:ti,ab,kw

#5 #3 and #4

Additional File 1. Medical Literature Search Results for Randomized Controlled Trials of Enteric-Coated Peppermint oil for the Irritable Bowel Syndrome.

Cochrane Library (60)

#1 MeSH descriptor: [Irritable Bowel Syndrome] explode all trees

#2 "irritable colon" or "irritable bowel syndrome" or "irritable bowel syndromes" or "Mucous Colitis" or "spastic colon" or "irritable colon" or "functional bowel" or "colonic disease" or "colonic diseases" or IBS or "gastrointestinal syndrome" or "gastrointestinal syndromes" or fgid or "functional gastrointestinal disorders" or "Colon spasm" or "Irritable colon syndrome" or "spastic colitis" or "unstable colon":ti,ab,kw

#3 #1 or #2

#4 "Peppermint oil" or "oleum menthae piperitae" or "peppermint essential oil" or mintoil or colpermin or "oil of peppermint" or "mentha piperita oil" or mintec:ti,ab,kw

#5 #3 and #4
