# Supplementary material for: The impact of peppermint oil on the irritable bowel syndrome: a meta-analysis of the pooled clinical data
Source: BMC Complement Altern Med. 2019 Jan 17;19:21. doi: 10.1186/s12906-018-2409-0 (PMC6337770; doi:10.1186/s12906-018-2409-0)
Supplement: Supplementary file 2 — Table of Excluded Studies. Ten of twenty-two included randomized controlled trials (RCTs) were eliminated for reasons shown. Five studies for data that was shown in other publications, four RCTs were cross-over trials without sufficient washout period, and an RCT was permitted rescue medication without sufficient data to permit analysis. (PDF 369 kb) [file 12906_2018_2409_MOESM2_ESM.pdf]

Additional File 2. Table of Excluded studies.

1. Cross-over design without a washout period, non-validated inclusion criteria, insufficient data.(1)
2. Cross-overdesign without a washout period, non-validated inclusion criteria.(2)
3. Cross-over design without a washout period, non-validated inclusion criteria.(3)
4. Insufficient data as subjects used rescue therapy without disclosing details. (4)
5. Cross-over design with a variable washout period, Insufficient data (conference abstract).(5)
6. Data presented elsewhere.(6)
7. Data presented elsewhere.(7)
8. Data presented elsewhere.(8)
9. Data presented elsewhere.(9)
10. Data presented elsewhere.(10)

1. Evans B**.** Multicentre trial of peppermint oil capsules in irritable bowel syndrome.[abstract]. Scand-J-Gastroenterol; 1982:503.
2. Lawson MJ, Knight RE, Tran K, Walker G, Robertsthomson IC**.** Failure of Enteric-Coated Peppermint Oil in the Irritable Bowel Syndrome - a Randomized, Double-Blind Crossover Study. Journal of Gastroenterology and Hepatology. 1988;3(3):235-8.
3. Nash P, Gould SR, Bernardo DE**.** Peppermint oil does not relieve the pain of irritable bowel syndrome. Br J Clin Pract. 1986;40(7):292-3.
4. Vejdani R, Shalmani HRM, Mir-Fattahi M, Sajed-Nia F, Abdollahi M, Zali MR, et al. The efficacy of an herbal medicine, Carmint, on the relief of abdominal pain and bloating in patients with irritable bowel syndrome: A pilot study. Digestive Diseases and Sciences. 2006;51(8):1501-7.
5. Rhodes J, Evans BK, Rees WDW**.** Peppermint oil in enteric coated capsules for the treatment of irritable bowel syndrome: A double blind controlled trial. Hepato-Gastroenterology. 1980;27(SUPPL.):E31.6.
6. Cash BD, Epstein MS, Shah S**.** Successful Management Viscerosensory Symptoms in Patients With IBS-M and IBS-D Using a Targeted Delivery System of Peppermint Oil (PO-SST). American Journal of Gastroenterology. 2015;110:S745-S.
7. Epstein MS, Cash BD, Shah S**.** Rapid relief of irritable bowel syndrome (IBS) symptoms with targeted delivery of l-menthol to the small intestine: Results from 2 clinical trials and a patient survey. American Journal of Gastroenterology. 2015;110:S741.
8. Cash B, Epstein M, Shah S**.** IBgard, a novel small intestine targeted delivery system of peppermint oil, results in significant improvement in severe and unbearable IBS symptom intensity. Results from a US based, 4-week, randomized, placebo-controlled, multi-center IBSRESTTM trial. Gastroenterology; 2015:S489.
9. Cash BD, Epstein MS, Shah SM**.** Ibgard (R), a Novel Targeted Delivery System of Peppermint Oil, Results in Significant Improvement in the Total IBS Symptom Score and Individual IBS Symptoms. Results From the US Based, 4-Week, Randomized, Placebo Controlled, Multi-Centered Ibsrest (TM) Trial. Gastroenterology. 2015;148(4):S662-S3.
10. Cappello G, Spezzaferro M, Coraggio D, Grossi L, Marzio L**.** Peppermint oil (Mintoil (R)) in the treatment of irritable bowel syndrome: A prospective double blind placebo controlled randomized trial. Gastroenterology. 2006;130(4):A322-A3.
